# Sensitivity of musculoskeletal models to variation in muscle architecture parameters

**DOI:** 10.1017/ehs.2022.6

**Published:** 2022-02-15

**Authors:** Patricia Ann Kramer, Elen M. Feuerriegel, Steven G. Lautzenheiser, Adam D. Sylvester

**Affiliations:** 1 Department of Anthropology, University of Washington, Denny Hall, Seattle, WA 98195, USA; 2 Evolutionary Studies Institute, University of Witwatersrand, Private Bag 3, Wits, 2050, South Africa; 3 Department of Anthropology, The University of Tennessee, Knoxville, Strong Hall, Knoxville, TN 37996, USA; 4 Center for Functional Anatomy and Evolution, The Johns Hopkins University School of Medicine, 1830 E. Monument Street, Baltimore, MD 21205, USA

**Keywords:** Human walking, muscle volume, muscle optimal fibre length

## Abstract

Musculoskeletal models, like all theoretical models of physical processes, depend on the assumptions needed to construct the model. For musculoskeletal models, these assumptions include, among other things, the kinematic data, the kinetic data and the muscle parameters. The former (dynamic) data can be acquired relatively easily from living subjects, but the latter are usually based on limited information, frequently determined from cadaver studies performed on elderly individuals. Previously, we determined the sensitivity of forces to dynamic differences among 10 humans walking on a straight path. Here, we assess the sensitivity of the muscle and joint reaction forces developed in human walking to variable muscle parameters obtained from 10 living adults, whose data were recently reported, and compared the results with the values from a standard model that depends on cadaveric data. We found that, while the force patterns across the stance cycle were similar among muscle parameter models, differences of as much as 15% in the force magnitude were produced. Whether or not the variation between the standard model and other muscle parameters is important depends on why the forces are required.

## Graphical abstract

**Figure:**
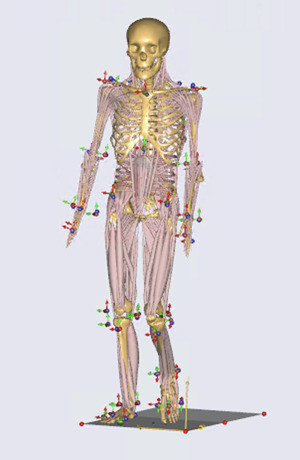

**Social media summary:** Typical human variation in muscle architecture changes internal forces by 15% in a musculoskeletal model of walking

The nature of the biomechanical requirements and consequences of bipedalism, from its inception in the hominin lineage to its manifestation in modern humans, remains a puzzle that motivates the research of many biological anthropologists, anatomists and functional morphologists. Bipedalism is rare among mammals, but ubiquitous within hominins for at least 4 million years (Harcourt-Smith, 2010), and because modern humans are the only remaining habitual, terrestrial bipedal primate, much work has centred on the bipedalism of modern humans. Insights into how human movement influences the musculoskeletal system has been extrapolated onto the movements of extinct hominins (e.g. Kramer & Eck, 2000; Lovejoy, 1988; Ruff & Higgins, 2013; Sellers, Cain, Wang, & Crompton, 2005). Inherent in this approach, however, is the notion of a ‘generic’, i.e. representative, model of humans, human musculoskeletal systems and human movements. In order to determine the limitations of such a generic approximation, the impact of human variability on the outcome of any particular analysis should be examined.

Our ultimate goal is to assess the impact of the forces generated in walking on the bones of the lower limb. Given that there is no one point in stance where every joint reaction and muscle force is at its maximum (Sylvester, Lautzenheiser, & Kramer, 2021b), we seek to develop a reduced set of generic conditions (each of which represents a point in the stance cycle) that are likely to be relevant to bone morphology. Here, ‘generic’ refers to conditions that are representative of humans, rather than a specific human. This reduced set of conditions that represents many points during stance allows us to create an envelope of cases. We can do this by identifying the points in the stance phase where the applied forces are maximum and whether or not these points are consistent across the stance phases within and among people. A key issue in this approach, however, is that the conditions are representative of humans. Consequently, we are critically concerned about human variability in kinematics and kinetics (motion and forces) and in musculoskeletal anatomy. Models are necessary because empirically measuring muscle forces in living animals depends on the surgical implantation of sensors into the body, which is invasive and expensive, and requires specialised equipment and personnel (e.g. Bey & Derwin, 2012; Pedersen, 1997).

Models are, then, approximations that are designed to represent phenomena that are difficult or impossible to investigate directly. Although models are useful to begin to gauge processes that cannot be empirically investigated, all such approximations have assumptions inherent in their development. Assessing the influence of these assumptions on outcome variables is critically important. Using kinematic and kinetic information from 10 individuals, we have evaluated the muscular forces among individuals (Sylvester et al., 2021b) and found that the functional muscle groups that are critically important to propel (i.e. the ankle plantarflexors) and stabilise (e.g. the hip abductors) the body are consistent among individuals and steps. Inherent in our use of this musculoskeletal model, however, were many assumptions, including those about the values of muscle volumes and optimal fibre lengths. In this work, we seek to determine the degree to which joint reaction and muscle forces of the lower limb vary with different muscle parameters.

Muscle forces are difficult to determine in musculoskeletal models because they exist in functional groups that are multiply redundant with respect to the joint of interest, meaning that multiple muscles can produce forces that create moments that act at particular joints and about particular axes. For example, soleus, plantaris and medial and lateral gastrocnemius all produce ankle plantarflexion. Consequently, the only way to resolve individual muscle forces (i.e. to solve the muscle redundancy problem) is to assume how the body allocates muscle force or, in other words, how the body determines the degree of active contraction to produce in each muscle at any given point. This physical system is difficult to study empirically, so modern musculoskeletal models use muscle strength (derived from muscle parameters such as fibre length, muscle volume, and pennation angle) to determine activation level via an allocation or cost algorithm. This allocation algorithm is the critical assumption for determining the force produced by a particular muscle because muscles in a functional group are all capable of producing forces that produce similar joint moments. Given that our goal is to use muscle forces to interpret bone morphology, the force that a particular muscle produces is critical because some muscles in a functional group originate or insert on different bones than other muscles in the same functional group. For example, soleus connects the tibia to the calcaneus across the ankle and subtalar joints, but medial and lateral gastrocnemius and plantaris originate on the femur, crossing subtalar, ankle and knee joints. Even though the primary action of all the muscles of the triceps surae complex is to cause the ankle to plantarflex, for examining femoral evolution, the distribution of force among the muscles is important because soleus does not directly interact with the femur.

Our musculoskeletal model produces muscular forces that are consistent among steps, trials and individuals for functional muscle groups (Sylvester et al., 2021b). We have, therefore, some understanding of the influence of variability in the kinematics and kinetics that is due to inter- and intra-individual differences on joint reaction and muscle forces. However, musculoskeletal models, including ours, are typically constructed using muscle architecture and geometry parameters drawn from either a single individual or small and morphologically homogeneous samples (e.g. Klein Horsman, Koopman, van der Helm, Prosé, & Veeger, 2007). Nonetheless, even for models of muscle function that are simpler than the Hill type, such as the muscle formation that we use, muscle architectures (e.g. optimal fibre length, muscle volume) are foundational inputs as they determine strength. The majority of reference datasets are drawn from elderly cadaveric specimens (e.g. Klein Horsman et al., 2007), on whom muscle dissection and examination are used to obtain the geometric parameters. These reference datasets are then used to construct generic musculoskeletal models which are assumed to be representative of the general population. How representative such limited samples are of the ‘average’ healthy adult remains an open question, however, as muscle architecture and geometry, including volume, fibre length and pennation angle, have been shown to change with age (Handsfield, Meyer, Hart, Abel, & Blemker, 2014; Narici, Maganaris, Reeves, & Capodaglio, 2003; Tate, Williams, Barrance, & Buchanan Thomas, 2006), among other individual characteristics.

Recently, Charles et al. (2019) used diffusion tensor magnetic resonance imaging and fibre tractography to visualise with a high degree of resolution the *in vivo* anatomy of a variety of muscle groups and to create one of the most comprehensive *in vivo* lower limb muscle architecture datasets available to date (Charles, Suntaxi, & Anderst, 2019). This novel dataset includes muscle architecture measurements from 20 lower limb muscles in 10 young, healthy individuals, with muscle volume, length, optimal fibre length, pennation angle, muscle physiological cross-sectional area and maximum isometric force provided for each subject. Continuing this work, Charles et al. (2020) compared the accuracy of both subject-specific lower limb models and generic models built using data from both elderly and young individuals with muscle values obtained experimentally using an isokinetic dynamometer (Charles, Grant, Août, & Bates, 2020). Their results demonstrated that, while the generic models were not as accurate as the subject-specific models in predicting the experimental muscle values of particular individuals, generic models still have utility in musculoskeletal modelling studies. One of the major limitations of this study was that the modelling predictions were only validated in comparison with muscle forces obtained in the sagittal plane involving one degree of freedom. It remains unclear how different subject specific and generic musculoskeletal models are for making functional predictions when simulating more complex movements such as gait.

Consequently, in this work we evaluate the impact of variability in muscle parameters and the choice of exponent in the cost algorithm on muscle functional groups of the lower limb that have both uniarticular and multiarticular muscles. Muscle functional groups are characterised by their primary function, although individual muscles within the group can have secondary functions. We identify the following functional muscle groups: the triceps surae (plantaris, soleus, and medial and lateral gastrocnemius), the hamstring muscles (semitendinosus, semimembranosus, biceps femoris caput breve, and biceps femoris caput longum) and the quadriceps (vastus lateralis, vastus intermedius, vastus medialis, and rectus femoris). Changing muscle parameters within a model does not change the external kinematic and kinetic conditions, so joint moments do not change. However, because muscle parameters determine muscle strength and the cost algorithm distributes force to muscles based on their strength, the distribution of force among the various muscles in a muscle functional group or among groups could change and this could change joint reaction forces. Consequently, although our approach is predominantly data exploration, we hypothesise that:

1. The shape of the joint reaction and muscle force profiles with respect to stance time will be similar among muscle parameter models in the same individual and among all individuals.
2. The distribution of maximum force within a functional group will vary based on strength of the individual muscles relative to the other muscles in that functional group.

## Materials and methods

### Dynamic cases: participants and protocol

Ten healthy participants (five female, five male; age 21–55 years; body weight 53.1–98.5 kg; stature 1.59–1.82 m) were recruited from the student population at the University of Washington. Exclusion criteria included gait abnormalities and a recent history of injury. Biometric data were collected from each participant, including stature, mass, limb segment length and limb segment circumference. The University of Washington's Institutional Review Board approved all aspects of this study (IRB no. STUDY00001125). The experimental protocol for the kinematic data collection process has been described in detail elsewhere (Sylvester, Lautzenheiser, & Kramer, 2021a, 2021b). We refer to the motion and force data of these participants as dynamic cases to distinguish them from subjects whose muscle parameters were used in the simulations described below.

The participants walked at their self-selected normal velocity across four force plates (Kistler, Switzerland) while the motions of 30 infrared-reflective markers placed on anatomical landmarks were measured with a 10 camera motion capture system (Qualisys, Sweden) in the Amplifying Movement and Performance Laboratory at the University of Washington. Each participant completed five trials, but the data from two trials for two participants were unusable, resulting in 46 total dynamic cases with two consecutive stance periods (92 total steps).

#### Baseline Musculoskeletal Model

The baseline model used to calculate lower limb joint reaction and muscle forces in this study was the MoCap model included as part of the commercially available AnyBody Modelling System (v. 7.3, AnyBody Technology, Denmark) and hosted on the AnyBody musculoskeletal model repository (AnyBody Managed Model Repository AMMR v. 2.3.0). This is a validated, multi-trial, full-body, motion-capture-driven human gait model with head, trunk, and left and right lower limb components (TLEM 2.0; De Pieri et al., 2018). The muscle architecture and geometry data for the baseline model were drawn from a single cadaveric individual, described in detail in Klein Horsman et al. (2007). We refer to this baseline MoCap model as the KH model.

Each lower limb of the KH model comprises thigh, patella, shank, talus and foot segments, and has six total degrees of freedom including all three rotations at the hip and one each at the knee (flexion/extension), ankle (plantarflexion/dorsiflexion) and subtalar (inversion/eversion) joints. The pelvis (relative to the ground) has six degrees of freedom, three translational and three rotational. Thirty-seven anatomical muscles are represented by 169 muscle elements (force actuators) in each lower limb (e.g. gluteus medius is composed of 12 separate muscle element actuators).

As detailed in our previous reports using these dynamic cases (Sylvester et al., 2021a, b), after initial scaling to the participant's body mass and stature, segment lengths are further adjusted to improve the match of the model with the motions described by the markers. This results in an individualised model for each participant based on their anthropometrics and motions. Then, an inverse dynamics solution of the individualised model is obtained for each trial and the muscle forces are allocated. Each trial produces a set of joint reaction and muscle forces. Variation within an individual is due to slight differences in motions or ground reaction forces, while variation among individuals includes differences in anthropometrics as well as the kinetic and kinematic variation. Thus, each dynamic model is ‘subject-specific’ for those input parameters.

We use a muscle recruitment algorithm which seeks to minimise the sum of the ratios of each muscle force to its strength raised to a power:1
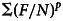
where *F* is the muscle force, *N* is the muscle strength and *p* is the power. Both *N* and *p* are model assumptions. We used the strength algorithm with an exponent of 3 as the baseline, consistent with our previous work (Sylvester et al., 2021a, b) and others (e.g. Simonsen et al., 2021). We also completed a set of simulations using a strength algorithm with an exponent of 2. We distinguish these versions of the same musculoskeletal model with KHP3 and KHP2, respectively.

### New muscle parameter model

The data from the 10 participants (Table 1) from Charles et al. (2019) were used to create MoCap models with revised muscle parameters. We refer to these models as CS1, CS2, and so on, where the number indicates the Charles et al. (2019) subject ID. Two main parameters that influence muscle force generation were identified as of interest for our simulations: optimal fibre length (*L*_opt_; Table 2) and muscle volume (*muscvol*; Table 3). To create the 10 new MoCap models modified to include muscle architecture parameters drawn from Charles et al. (2019), we adjusted the parameters of interest to the size of the KH model. Specifically, to adjust muscle volume, we summed the total volume of muscle for the KH model based on the published values provided for the KH cadaveric specimen (Klein Horsman et al., 2007). We then calculated the ratio of muscle volume for each muscle and muscle element (proximal, mid and distal; inferior and superior; medial and lateral depending on the muscle) to the total muscle volume in the Charles et al. (2019) subjects and multiplied by the total muscle volume of the KH baseline model. CS muscle volume in model = (CS muscle volume/CS total muscle volume) × KH total muscle volume.

**Table 1.** Characteristics of subjects with muscle parameter data (Charles et al., 2019)

Muscle parameter case	Sex	Body mass (kg)	Stature (cm)	Graph colour	Closest match to dynamic subject
CS1	M	90.7	182	Red	2
CS2	M	82.1	173	Green	4
CS3	M	81.1	182	Blue	9
CS4	F	71.2	162	Magenta	6
CS5	F	59.8	170	Green	6
CS6	F	80.2	169	Cyan	5
CS7	F	80.7	168	Red	5
CS8	F	40.6	162	Blue	1
CS9	M	84.8	187	Cyan	9
CS10	M	82.5	192	Magenta	2

**Table 2.** Optimal fibre lengths (mm)

			Adjusted optimal fibre length									
Muscle	Segment	KH	CS1	CS2	CS3	CS4	CS5	CS6	CS7	CS8	CS9	CS10
Adductor magnus	Distal	107.80	234.44	303.97	278.66	133.63	170.00	238.70	298.98	109.83	276.80	259.25
	Mid	104.30	234.44	303.97	278.66	133.63	170.00	238.70	298.98	109.83	276.80	259.25
	Proximal	87.00	234.44	303.97	278.66	133.63	170.00	238.70	298.98	109.83	276.80	259.25
Adductor longus		10.57	104.88	122.18	107.97	46.68	139.27	129.85	132.88	70.47	118.33	134.51
Adductor brevis	Proximal	95.30	62.72	101.65	75.06	31.12	96.05	73.52	77.83	48.51	104.59	97.63
	Mid	103.80	62.72	101.65	75.06	31.12	96.05	73.52	77.83	48.51	104.59	97.63
	Distal	112.20	62.72	101.65	75.06	31.12	96.05	73.52	77.83	48.51	104.59	97.63
Gracilis		181.10	232.38	153.45	217.99	160.17	104.69	148.95	124.34	67.73	277.86	241.90
Semimembranosus		80.90	107.97	166.16	192.28	116.24	169.04	184.28	105.36	104.34	260.95	164.88
Semitendinosus		141.60	173.77	96.76	162.46	208.68	227.63	203.37	143.32	122.64	246.16	226.71
Biceps femoris – long head		85.40	131.62	185.71	219.02	220.58	190.17	200.51	137.63	216.92	225.03	224.54
Biceps femoris – short head		91.40	110.02	73.31	162.46	125.39	102.77	112.67	174.64	75.05	115.16	107.39
Popliteus		24.00	76.09	56.69	97.68	50.34	90.28	57.29	82.58	66.81	79.24	70.51
Sartorius		347.10	465.80	390.96	446.26	356.03	378.42	360.92	349.29	372.51	458.52	472.95
Rectus femoris		78.40	114.14	123.15	124.42	137.29	134.46	208.15	141.42	57.66	220.81	165.97
Vastus lateralis	Inferior	42.50	118.25	206.23	237.53	210.51	145.99	261.62	160.41	171.15	226.09	212.61
	Superior	91.20	118.25	206.23	237.53	210.51	145.99	261.62	160.41	171.15	226.09	212.61
Vastus medialis	Inferior	56.00	122.36	100.67	182.00	192.20	140.23	140.36	197.42	104.34	236.66	171.39
	Mid	75.80	122.36	100.67	182.00	192.20	140.23	140.36	197.42	104.34	236.66	171.39
	Superior	83.00	122.36	100.67	182.00	192.20	140.23	140.36	197.42	104.34	236.66	171.39
Vastus intermedius		76.80	187.14	125.11	148.07	196.78	158.47	217.69	203.12	105.25	239.82	233.22
Tibialis anterior		68.30	171.72	130.97	171.72	99.76	96.05	96.44	186.03	128.14	157.42	176.81
Extensor digitorum longus		59.90	176.86	100.67	130.59	118.98	97.01	129.85	145.22	130.88	193.34	136.68
Extensor hallucis longus		59.80	107.97	79.17	135.73	117.15	65.31	92.62	152.81	72.31	151.08	105.22
Medial gastrocnemius		60.10	81.23	86.01	107.97	75.05	85.48	115.53	125.29	63.15	153.19	111.73
Lateral gastrocnemius		56.90	147.04	72.33	149.10	67.73	122.94	151.81	79.73	80.54	198.62	180.07
Soleus	Medial	44.00	192.28	177.89	111.05	108.00	134.46	149.90	211.66	136.37	163.76	96.54
	Lateral	44.00	192.28	177.89	111.05	108.00	134.46	149.90	211.66	136.37	163.76	96.54

**Table 3.** Muscle volumes (ml)

			Adjusted muscle volume									
Muscle	Segment	KH	CS1	CS2	CS3	CS4	CS5	CS6	CS7	CS8	CS9	CS10
Adductor magnus	Distal	285.98	380.03	315.22	261.49	322.17	235.96	314.56	260.98	306.07	278.20	303.45
	Mid	230.11	305.78	253.63	210.40	259.23	189.87	253.11	210.00	246.27	223.85	244.17
	Proximal	43.16	57.35	47.57	39.46	48.62	35.61	47.47	39.39	46.19	41.99	45.80
Adductor longus		159.56	168.26	153.63	197.63	112.46	178.04	223.02	136.40	163.53	208.46	176.37
Adductor brevis	Proximal	36.30	30.01	31.73	27.50	24.48	40.37	78.57	33.72	35.98	27.11	32.85
	Mid	36.30	30.01	31.73	27.50	24.48	40.37	78.57	33.72	35.98	27.11	32.85
	Distal	36.30	30.01	31.73	27.50	24.48	40.37	78.57	33.72	35.98	27.11	32.85
Gracilis		87.97	104.06	76.35	95.94	87.22	99.31	91.96	91.96	70.32	123.18	85.07
Semimembranosus		138.26	202.21	222.44	247.52	300.66	259.18	284.32	245.22	253.48	286.63	262.47
Semitendinosus		208.33	200.74	182.85	247.52	176.73	215.58	149.03	185.45	179.89	198.98	189.85
Biceps femoris- long head		232.01	238.37	184.74	231.21	184.76	186.51	198.71	217.63	191.33	160.29	224.09
Biceps femoris- short head		107.95	98.89	72.58	104.57	104.43	93.26	71.87	139.47	112.84	99.49	78.85
Popliteus		25.57	19.19	15.08	18.23	13.77	13.32	15.85	15.33	13.08	16.58	10.37
Sartorius		205.49	140.22	125.36	143.91	193.94	136.86	143.75	197.71	112.84	167.40	129.68
Rectus femoris		226.33	237.64	232.81	306.04	246.73	330.64	214.56	234.49	251.84	276.36	264.55
Vastus lateralis	Inferior	45.45	51.75	47.37	49.93	57.05	52.18	43.81	49.32	50.07	48.91	42.19
	Superior	537.88	612.44	560.57	590.93	675.10	617.57	518.49	583.66	592.61	578.83	499.35
Vastus medialis	Inferior	54.65	56.64	57.04	47.44	55.77	48.37	47.68	46.10	54.50	52.82	50.67
	Mid	176.14	182.57	183.83	152.89	179.76	155.91	153.69	148.57	175.64	170.23	163.32
	Superior	223.48	231.63	233.23	193.98	228.08	197.81	194.99	188.50	222.85	215.98	207.21
Vastus intermedius		292.61	537.26	597.57	572.75	539.36	574.08	551.73	455.19	560.92	461.92	579.93
Tibialis anterior		181.49	107.75	140.44	146.79	109.02	157.45	134.23	179.32	140.64	119.23	141.09
Extensor digitorum longus		32.29	56.09	87.66	69.08	64.26	106.58	73.99	76.63	99.76	80.54	97.52
Extensor hallucis longus		36.27	21.40	30.16	23.98	22.95	6.06	19.03	32.19	24.53	15.79	21.79
Medial gastrocnemius		263.26	209.59	223.38	222.58	219.19	244.65	263.18	332.58	210.96	245.57	257.29
Lateral gastrocnemius		136.36	104.06	156.46	120.88	146.89	122.32	127.89	142.54	112.84	155.55	143.17
Soleus	Medial	414.86	218.94	281.09	237.93	214.95	237.63	240.56	273.45	278.08	274.32	269.77
	Lateral	378.05	199.51	256.15	216.82	195.88	216.54	219.22	249.18	253.40	249.98	245.84

To scale optimal fibre length (*L*_opt_), a similar process was undertaken using stature: the stature of the KH model was divided by the stature of the Charles et al. subject and multiplied by the optimal fibre length values provided in Charles et al. (2019) for that subject. As Charles et al. (2019) does not distinguish among the muscle regions for all muscles included in the MoCap model (e.g. soleus is divided into medial and lateral portions in the MoCap model, but not in the Charles et al., 2019 data), we elected to use the same *L*_opt_ for each region.

The adjustment of these two muscle parameters generated 10 new, modified models – one model based on each of the 10 subjects from the Charles et al. (2019) study. Table 2 lists the optimal fibre length and Table 3 details the muscle volumes used for the baseline KH model and each of the modified CS models in this study.

### Model simulation

Each CS model was simulated using the gait kinematic data drawn from each of the 10 participants in the experimental walking protocol described by Sylvester et al. (2021b), resulting in 460 simulations. To this dataset were added the baseline muscle parameter models (KHP3 and KHP2), resulting in a total of 552 simulations and 1104 steps. All CS simulations used an exponent of 3 in the strength algorithm.

### Sensitivity analysis

We created three muscle functional groups that contain both uniarticular and multiarticular muscles that cross the knee (Table 4). We visually assessed the patterns of the joint reaction forces and muscle forces within dynamic cases for all muscle parameter models and among individual averages for all muscle parameter models for the functional muscle groups. We determined the strength of the functional muscle groups and the uni- and multiarticular portions by summing the strengths of the muscle elements of the group. The strength ratio of each functional muscle group is the strength of the uniarticular muscles divided by the multiarticular muscles. We determined the point in the stance of the maximum total muscle force of a functional group and determined the ratio of the force of the uniarticular muscle(s) to that of the multiarticular muscle(s) at that point.

**Table 4.** Definition of muscle functional groups

Muscle functional group	Uniarticular muscles	Multiarticular muscles
Triceps surae	Soleus	Medial gastrocnemius Lateral gastrocnemius Plantaris^a^
Hamstrings	Biceps femoris short head	Biceps femoris long head Semitendinosus Semimembranosus
Quadriceps	Vastus lateralis Vastus intermedius Vastus medialis	Rectus femoris

a Plantaris is not traditionally included in triceps surae, but we include it here as it has similar function to soleus and gastrocnemius.

Although our focus here is on data exploration – e.g. whether the joint reaction and muscle force profiles are consistent – to address hypothesis 2, we predicted the maximum force ratio from the strength ratio in linear and quadratic forms, using linear regression with correction for repeated measures (i.e. the dynamic cases; Stata, StataCorp, College Station, TX USA), to gain insight into how the muscle force allocation algorithm influences the joint reaction and muscle forces.

## Results

**Figure 1.**
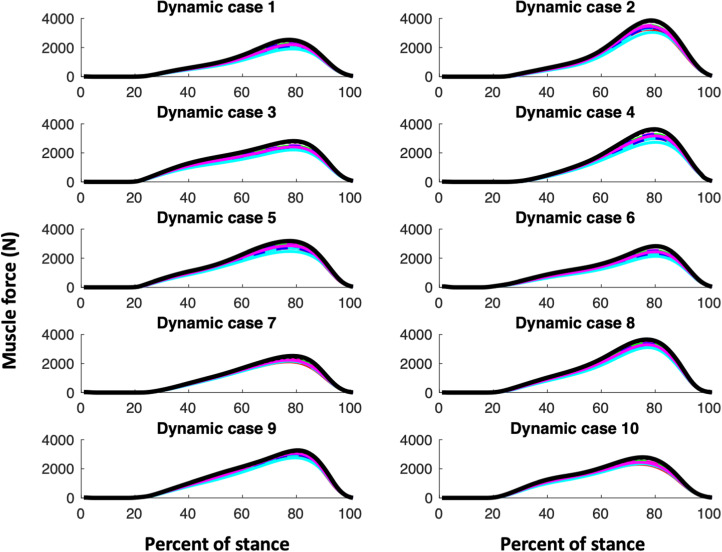
Force in triceps surae. For the CS subjects, males are indicated with a solid line while females are indicated with a dashed line. The colour of the line indicates the CS subject (Table 1): CS1 and CS7 are shown in red; CS2 and CS6 are shown in green; CS3 and CS8 are shown in blue; CS4 and CS10 are shown in magenta; CS6 and CS9 are shown in cyan. KHP3 is shown with a solid black line, while KHP2 is indicated with a dotted black line.

**Figure 2.**
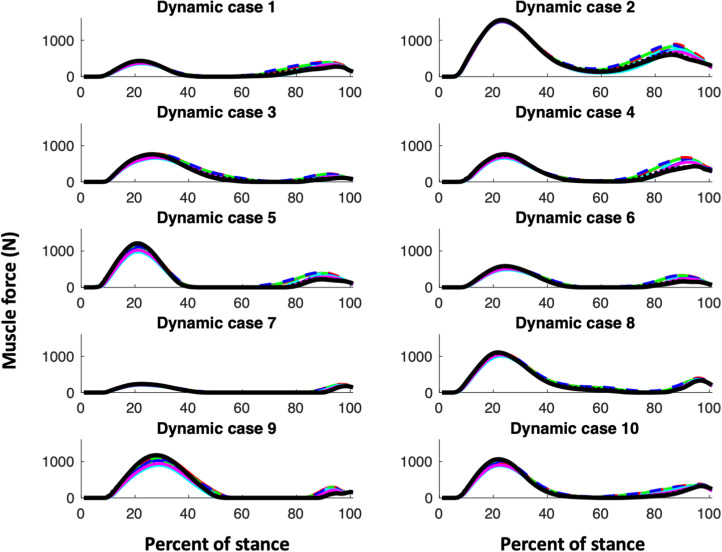
Force in quadriceps. For the CS subjects, males are indicated with a solid line while females are indicated with a dashed line. The colour of the line indicates CS subject (Table 1): CS1 and CS7 are shown in red; CS2 and CS6 are in green; CS3 and CS8 are in blue; CS4 and CS10 are in magenta; CS6 and CS9 are in cyan. KHP3 is shown with a solid black line, while KHP2 is indicated with a dotted black line.

**Figure 3.**
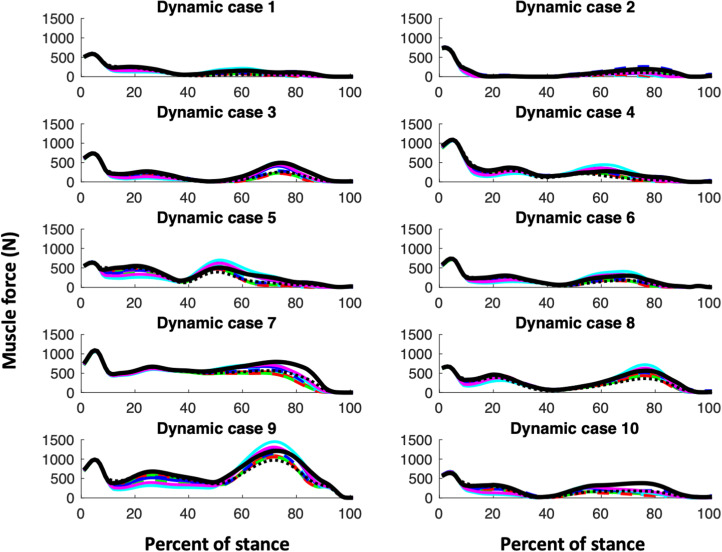
Force in hamstrings. For the CS subjects, males are indicated with a solid line while females are indicated with a dashed line. The colour of the line indicates the CS subject (Table 1): CS1 and CS7 are shown in red; CS2 and CS6 are shown in green; CS3 and CS8 are shown in blue; CS4 and CS10 are shown in magenta; CS6 and CS9 are shown in cyan. KHP3 is shown with a solid black line, while KHP2 is indicated with a dotted black line.

**Figure 4.**
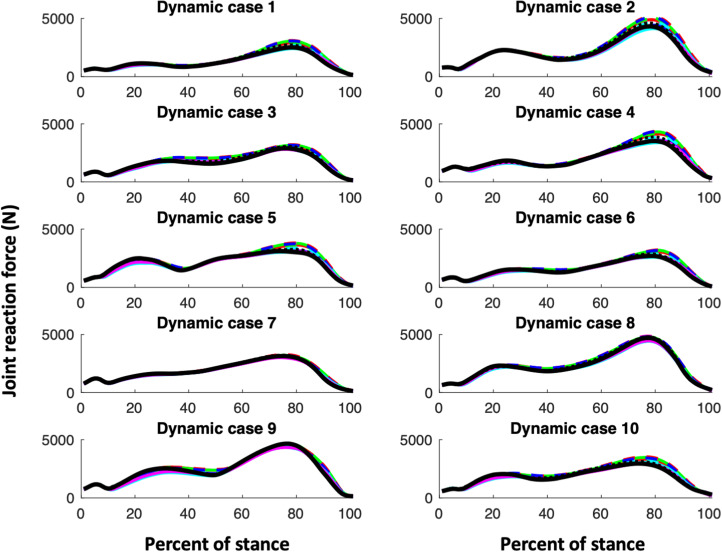
Knee joint forces. For the CS subjects, males are indicated with a solid line while females are indicated with a dashed line. The colour of the line indicates the CS subject (Table 1): CS1 and CS7 are shown in red; CS2 and CS6 are shown in green; CS3 and CS8 are shown in blue; CS4 and CS10 are shown in magenta; CS6 and CS9 are shown in cyan. KHP3 is shown with a solid black line, while KHP2 is indicated with a dotted black line.

Our first hypothesis is that muscle parameter models, i.e. the CS models and KH, would all exhibit similar shapes of their joint reaction and muscle functional group force curves. Figures 1–3 (muscle forces for the triceps surae, quadriceps and hamstring muscle functional groups, respectively) and Figure 4 (magnitudes of the knee joint reaction force; similar figures for the hip and ankle are provided in the Appendix, Figures A1 and A2) demonstrate that our expectations are broadly confirmed for each dynamic case. The muscle forces of the triceps surae group (Figure 1) and the joint reaction forces (Figure 4, A1 and A2) are consistent in pattern within a dynamic case and among them. The triceps surae force peaks in late stance (~80% of stance) and the joint reaction forces also exhibit peaks around 80% of stance. Of note, however, is that the shapes of the curves, while similar among muscle parameter models within a dynamic case, can vary considerably among dynamic cases. The muscle forces of the quadriceps group peak in early stance (~20% of stance) but dynamic case 7 (< 250N) exhibits much lower force peak than do cases 2 or 9 (> 1000N; Figure 2). The shapes of hamstring muscle functional group (Figure 3) demonstrate this variability: in dynamic cases 8 and 9 a distinct peak in hamstring force at 70–80% of stance is clear, while no such peak is apparent in dynamic case 1. Additionally, while the shapes of the curves are consistent, the difference in joint reaction force magnitudes within a dynamic case for the various muscle parameter models can exceed 700N or greater than 15% (e.g. hip forces for dynamic case 9; Figure A1).

**Figure 5.**
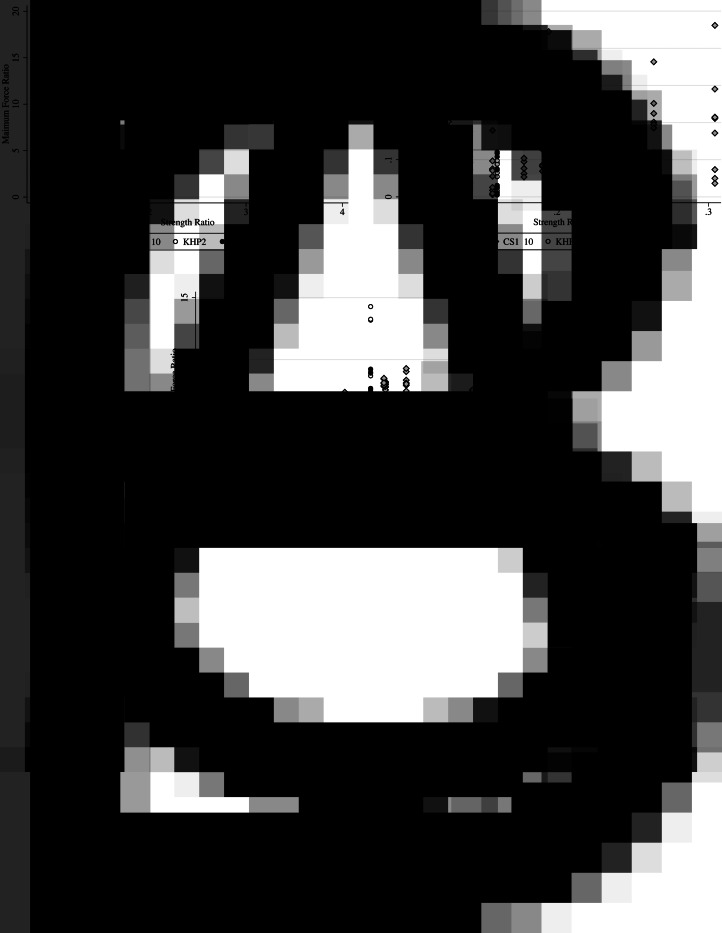
Peak muscle forces vs. strength in triceps surae: (a), quadriceps (b) and hamstrings (c).

The second hypothesis, that variation in muscle strength predicts variation in the distribution of the forces to bi- and uniarticular muscles, was not rejected. Figure 5 demonstrates the relationship between peak muscle force ratio and strength ratio for the three muscle functional groups. In triceps surae, CS and KHP3 follow a strong trend (*r*^2^ = 0.91, *p* < 0.001). In the hamstring muscles, CS and KHP3 also follow a strong trend (*r*^2^ = 0.71, *p* < 0.001). In the quadriceps muscles, CS and KHP3 demonstrate the same trend (*r*^2^ = 0.12, *p* = 0.002; Figure 5).

The KH muscle parameter model with an exponent of 2 for the cost equation can change the hip and knee joint reaction forces relative to using an exponent of 3 with slightly higher knee, but slightly lower hip forces (Figures 4 and A1). The effect of the exponent on the ankle is minimal (Figure A2). An exponent of 2 produces lower triceps and hamstring muscle forces, but higher quadriceps forces (Figures 1–3). An exponent of 2 produces a different relationship between uni- and multiarticular muscles than has an exponent of 3 for triceps and hamstring muscles, but not for quadriceps (Figure 5).

## Discussion

Our ultimate goal is to produce a generic set of joint reaction and muscle forces that can be used to interrogate the effect of variation in bone morphology that is seen in the fossil record. We had previously found consistency within and among individuals in the forces exerted by the propulsive and stability-focused muscle functional groups when we used the same muscle parameters (Sylvester et al., 2021b). Consequently, we conducted the current study to evaluate the impact of varying muscle parameters on the joint reaction and muscle forces and found that the shapes of the curves, especially within a dynamic subject, are similar, although the forces within a subject can vary among muscle parameter models.

The triceps surae muscle force for the KHP3 muscle parameter model is consistently greater than that for all of the CS muscle parameter models. Interestingly, the KH muscle parameter model has very large muscle volume values for soleus compared with the CS muscle parameter models: medial segment = 414.8 ml; lateral segment = 378 ml in KH vs. medial segment = 214.9–281 ml; lateral segment = 195.8–256.1 ml in the CS models. Gastrocnemius muscle volumes are more similar between KH and CS muscle parameter models. Because muscle volume is a contributor to muscle strength and because muscle strength determines the distribution of force among the available muscles, the larger soleus muscle volume in the KH model potentially draws more force to the soleus in the muscle redundancy algorithm. Similarly, the smaller soleus muscle volumes of the CS models cause the algorithm to allocate more muscle force to the medial and lateral gastrocnemius. In the KH models where soleus is larger (higher strength) and, therefore, is allocated more force, the soleus's somewhat shorter moment arm requires a higher muscle force, which increases joint reaction forces of the ankle (Figure A2).

The muscle forces in the quadriceps group in the KHP3 model are similar to, or somewhat higher than, the CS muscle model parameter models in the earlier parts of the stance phase, but smaller in the later portion of stance. The hamstring group demonstrates a more complex picture that depends on the portion of stance and the particular CS muscle parameter model. The knee joint forces also depend on the muscle parameter model to some degree with the KH model producing lower and higher forces than the CS model in some dynamic cases. As with all models, assumptions are necessary and whether or not the differences in magnitudes that we found are important depends on the question of interest. To demonstrate this point we offer two possible analyses that might utilise these data.

First, our proximate goal is to create an envelope of boundary conditions in a finite element model in order to interrogate the impact of localised morphology, e.g. anteroposterior flattening of the femoral neck in *Australopithecus* on femoral neck strain, during bipedal walking. Each muscle insertion or origin and the joint contact surfaces of the bone actively load the bone across the stance phase. Each boundary condition constitutes a non-trivial exercise in pre- and post-processing the finite element model. Our initial focus is the hominin femur; our goal, therefore, is to identify those points in the stance phase likely to produce the highest strains in the femur. Consequently, we aim to reduce the number of conditions to an envelope that contains as few conditions as possible to produce the maximum strains in the femur. We specifically seek to identify the points in the stance cycle when hip, knee or patellar joint reaction or femoral muscle forces are maximum within and among people given inherent kinematic, kinetic and muscular variability. While the creation of this envelope is beyond the scope of this paper, it is clear that conditions in early (~20% of stance phase) and late (~80%) stance are needed due to the peaks in the hip and knee joint contact and muscle forces that occur during those gait phases and that conditions near midstance might be less useful. Most biomechanical analyses of the evolution of the morphological features associated with bipedalism have focused on a single stance (Lovejoy, 1988; Ruff & Higgins, 2013), but our current work indicates that more attention needs to be directed towards the loading response (immediately after foot flat) and terminal stance (as the heel loses contact with the substrate) phases of gait. Additionally, our results suggest that it is possible to construct a ‘generic human’ model. The triceps surae functional group, the main source of propulsion in human bipedalism, demonstrates a consistent muscle force pattern across muscle models (as demonstrated here; Figure 1) and among individuals.

A second example of how these data might be used involves the variations in knee joint contact forces (Figure 4) that arise owing to the activation level of the muscle of triceps surae. Our results suggest that researchers interested in knee function in modern humans should focus attention on the influence of muscle parameters on the force distribution among the muscles of the triceps surae complex. Because triceps surae inserts on the calcaneus via the Achilles tendon, the cost algorithm solution for the ankle plantarflexion moment ‘sees’ all of the anatomical muscles of the complex as similar. Soleus does not, however, cross the knee, while gastrocnemius and plantaris do. The impact of triceps surae on lower limb biomechanics is well understood, as is the importance of gastrocnemius activation on knee contact force (e.g. Killen et al., 2021; Mengarelli et al., 2018). Nonetheless, our results demonstrate that knee joint forces increased when the muscle volume of soleus decreased because more propulsive force was distributed to medial and lateral gastrocnemius (which cross the knee), even though the patterns are consistent. Consequently, muscle parameter choices may be important determinants of joint forces and have relevance for such questions as the development of the femoral bicondylar angle in bipeds (Shefelbine, Tardieu, & Carter, 2002) or the differences in shapes of the medial and lateral condyles between australopiths and modern humans that are assumed to be caused by the distribution of forces (e.g. Preuschoft & Tardieu, 1996).

Beyond the utility of this analysis for specific questions, it seems clear more generally that the pattern among and within individuals for functional muscle groups with large magnitudes (e.g. for walking, triceps surae) is consistent when the muscle parameters (muscle volume and optimal fibre length) are varied, which is consistent with our previous work examining kinematic and kinetic variation (Sylvester et al., 2021b). Nonetheless, the magnitude of the forces may vary considerably (15%; Figure 1 and 4). Consequently, comparisons among morphological forms using the same external kinematics and kinetics and muscle model assumptions are preferable to analyses that focus on absolute magnitudes without control of kinematic, kinetic or muscle assumptions.

This simulation uses an algorithm based on muscle strength to resolve the joint moments (that are determined by the external kinematic and kinetic data) into the muscle forces, but this seemingly straightforward relationship is variably reflected in multiarticular muscles. While the relationship between relative maximum force and strength exhibits the shape expected from the algorithm for the triceps surae and hamstring functional groups (Figure 5), that of the quadriceps group is more variable.

A principal limitation of these analyses is that the kinematic and kinetic data derived from different individuals than the muscle parameter model data. Consequently, we do not know if the movements of the dynamic individuals could be achieved by each individual whose data were used to create the muscle parameter models. Most musculoskeletal model simulations use generic muscle parameter models, typically derived from elderly cadavers (e.g. Klein Horsman et al., 2007), so this limitation is not unique to this study. Additionally, we only varied muscle volume and optimal fibre length to create the muscle parameter models. While the Charles et al. (2019) data include pennation angle, we lacked the data to relate the orientations of the AnyBody muscle elements to the Charles et al. full muscles. We also did not address other areas of interindividual variation that could impact joint reaction and muscle forces, including variability in bone shape (e.g. pelvic shape (Cox, 2021), femoral torsion (De Pieri et al., 2021)), muscle origin and insertion locations (e.g. tibialis anterior (Zielinska et al., 2021), popliteus (Berthaume, Barnes, Athwal, & Willinger, 2020)), or even the presence or absence of anatomical elements (e.g. fabella (Berthaume & Bull, 2020), plantaris (Olewnik et al., 2020)). Finally, we chose to use a simplified muscle formulation rather than the more sophisticated Hill-type muscle because the Hill-type muscle formulation requires more detailed data than were available. Given that our approach is more focused on data exploration with the goal of developing an envelope of boundary conditions, this simpler model formulation is reasonable.

## Data Availability

The MoCap model is available from: https://anyscript.org/ammr-doc/index.html. The kinematics and kinetics are available from the Supplementary Information of Sylvester et al. (2021b). The muscle parameter changes are detailed in Tables 2 and 3.

## Supplementary material

For supplementary material accompanying this paper visit https://doi.org/10.1017/ehs.2022.6. click here to view supplementary material
