# Supplementary material for: Sensitivity of musculoskeletal models to variation in muscle architecture parameters
Source: Evol Hum Sci. 2022 Feb 15;4:e6. doi: 10.1017/ehs.2022.6 (PMC10426084; doi:10.1017/ehs.2022.6)
Supplement: Supplementary file 1 [file S2513843X22000068sup001.zip › supplement.docx]

**Figure A1.** Hip joint forces. For the CS subjects, males are indicated with a solid line while females are indicated with a dashed line. The colour of the line indicates the CS subject (Table 1): CS1 and CS7 are shown in red; CS2 and CS6 are shown in green; CS3 and CS8 are shown in blue; CS4 and CS10 are shown in magenta; CS6 and CS9 are shown in cyan. KHP3 is shown with a solid black line, while KHP2 is indicated with a dotted black line.

**Figure A2.** Ankle joint forces. For the CS subjects, males are indicated with a solid line while females are indicated with a dashed line. The colour of the line indicates the CS subject (Table 1): CS1 and CS7 are shown in red; CS2 and CS6 are shown in green; CS3 and CS8 are shown in blue; CS4 and CS10 are shown in magenta; CS6 and CS9 are shown in cyan. KHP3 is shown with a solid black line, while KHP2 is indicated with a dotted black line.
