# Supplementary figures and images for: Sensitivity of musculoskeletal models to variation in muscle architecture parameters
Source: Evol Hum Sci. 2022 Feb 15;4:e6. doi: 10.1017/ehs.2022.6 (PMC10426084; doi:10.1017/ehs.2022.6)

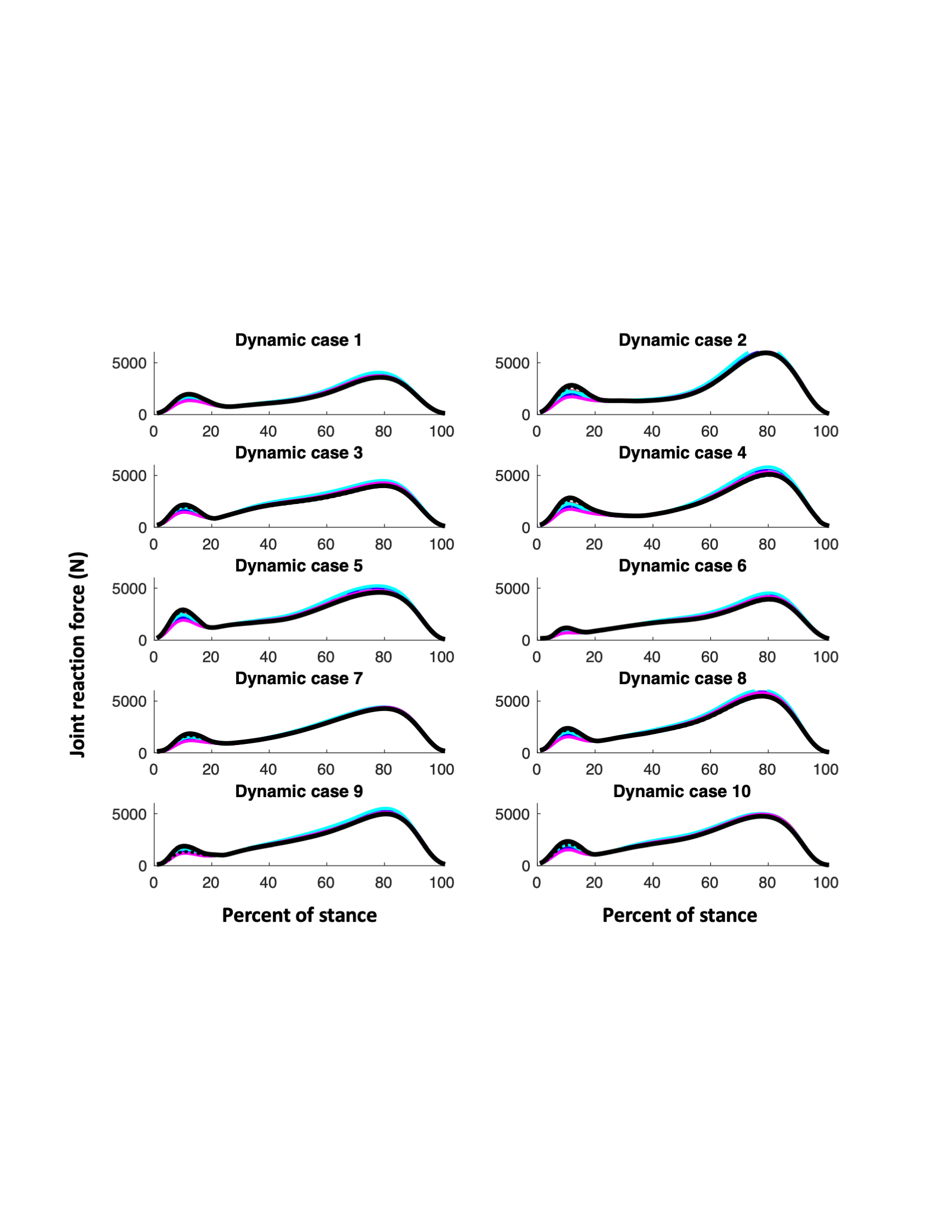

Supplement: Supplementary file 1 [file S2513843X22000068sup001.zip › figure A2.tiff]

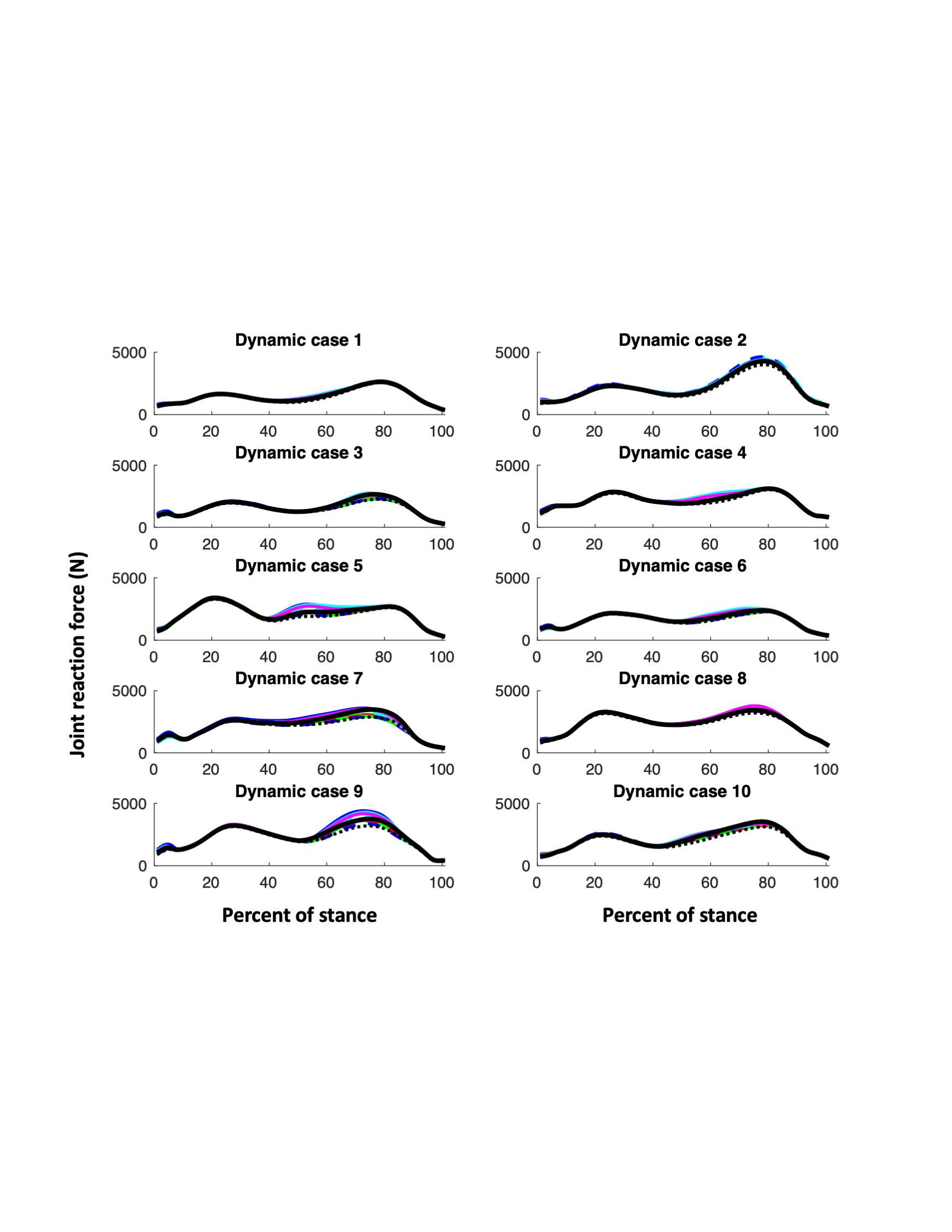

Supplement: Supplementary file 1 [file S2513843X22000068sup001.zip › figure A1.tiff]
